# Evidence of weak localization in quantum interference effects observed in epitaxial La_0.7_Sr_0.3_MnO_3_ ultrathin films

**DOI:** 10.1038/srep26081

**Published:** 2016-05-16

**Authors:** Wei Niu, Ming Gao, Xuefeng Wang, Fengqi Song, Jun Du, Xinran Wang, Yongbing Xu, Rong Zhang

**Affiliations:** 1 National Laboratory of Solid State Microstructures, Collaborative Innovation Center of Advanced Microstructures, and School of Electronic Science and Engineering, Nanjing University, Nanjing 210093, China; 2 National Laboratory of Solid State Microstructures, Collaborative Innovation Center of Advanced Microstructures, and Department of Physics, Nanjing University, Nanjing 210093, China

## Abstract

Quantum interference effects (QIEs) dominate the appearance of low-temperature resistivity minimum in colossal magnetoresistance manganites. The *T*^1/2^ dependent resistivity under high magnetic field has been evidenced as electron-electron (e-e) interaction. However, the evidence of the other source of QIEs, weak localization (WL), still remains insufficient in manganites. Here we report on the direct experimental evidence of WL in QIEs observed in the single-crystal La_0.7_Sr_0.3_MnO_3_ (LSMO) ultrathin films deposited by laser molecular beam epitaxy. The sharp cusps around zero magnetic field in magnetoresistance measurements is unambiguously observed, which corresponds to the WL effect. This convincingly leads to the solid conclusion that the resistivity minima at low temperatures in single-crystal manganites are attributed to both the e-e interaction and the WL effect. Moreover, the temperature-dependent phase-coherence length corroborates the WL effect of LSMO ultrathin films is within a two-dimensional localization theory.

The appearance of a low-temperature resistivity minimum has been observed in polycrystalline and single-crystalline colossal magnetoresistance (CMR) manganites^1^,^2^. Although great efforts were devoted to explaining the resistivity minima behavior in manganites, no convincing conclusions have yet been reached up to now. In the past few years, people have attributed the resistivity minima to different mechanisms, such as spin-polarized tunneling through grain boundaries^1^,^3^, Kondo-like effect due to spin disorder^4^, as well as quantum interference effects (QIEs)^5^. In polycrystalline samples, the resistivity minima at low temperatures shift towards the lower temperatures upon applying a magnetic field and disappear at certain critical fields, which is interpreted in terms of the spin-polarized tunneling via grain boundaries^1^,^6^,^7^,^8^. While Kondo effect dominates in intrinsically disordered samples^9^,^10^. Recently, QIEs in manganites have been intensively investigated to interpret the low-temperature resistivity minima^2^,^6^,^9^,^11^,^12^,^13^,^14^. Generally, QIEs lead to correction to the resistivity from two different sources^5^: (i) electron-electron (e-e) interaction and subsequent modification of the density of states at the Fermi energy; (ii) weak localization (WL) effect arising from the self-interference of the wave pockets as they are backscattered coherently by the impurities or other defects. Both contributions lead to an enhancement of resistivity as the temperature decreases. Previously, Li *et al*.^14^ investigated the temperature and field dependence of the conductivity of a La_1.2_Sr_1.8_Mn_2_O_7_ single crystal in terms of QIEs. Maritato *et al*.^2^ investigated the low-temperature transport properties of La_0.7_Sr_0.3_MnO_3_ (LSMO) films as a function of the sample thickness, and interpreted their results as an interplay of e-e interaction and WL effect. Gao *et al*.^12^ studied the resistivity minimum of LSMO films integrated with nonmagnetic ZrO_2_ particles as a second phase to tune the contribution of an enhanced three dimensional WL effect.

The *T*^1/2^ dependent resistivity under high magnetic fields has been evidenced as e-e interaction. Xu *et al*.^7^ investigated the behavior of the resistivity minima with various magnetic fields, and they found that the WL effect was suppressed by a high field (*H* > 1 T) and the electrical resistivity only followed the *T*^1/2^ dependence with the characteristics of enhanced e-e interaction. However, to date the evidence of WL effect has still remained insufficient in manganites. Although vast phenomenological analyses have been fitted to prove the existence of the WL effect^2^,^11^,^12^, the typical WL effect in magnetoresistance (MR) measurements, i.e., the sharp cusps around zero magnetic field at low temperatures, has not been observed. Thus, the conclusion that low-temperature resistivity minima are attributed to QIEs with the combination of e-e interaction and WL effect requires the further direct experimental evidence.

In this work, the low-temperature resistivity minima and transport properties of LSMO ultrathin films are investigated. The films are deposited by laser molecular beam epitaxy (LMBE) on SrTiO_3_ (STO) substrates. We give direct experimental evidences of e-e interaction and WL effect. The *T*^1/2^ dependent resistivity under high magnetic field is evidenced as e-e interaction. In particular, the sharp cusps around zero magnetic field in MR measurements is evidenced as the typical WL effect. This leads to the solid conclusion that the low-temperature resistivity minima in single-crystal manganites are the result of QIEs from the combination of e-e interaction and WL effect. Moreover, the power law fit of phase-coherent length versus temperature further confirms the WL effect of our ultrathin films is within a two-dimensional (2D) localization theory.

## Results

### High-quality LSMO ultrathin films

**Figure 1:**
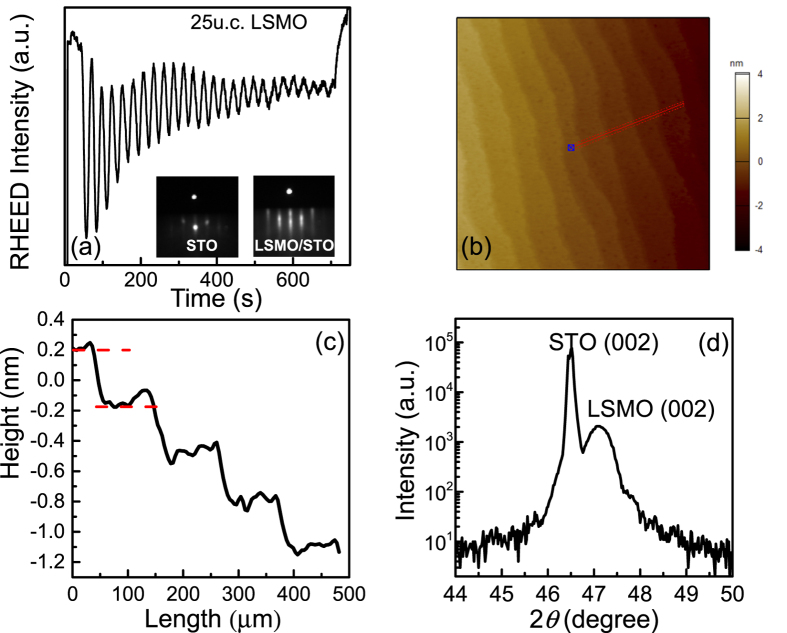
Structural analysis of epitaxial LSMO ultrathin films grown on STO. (**a**) Typical RHEED intensity oscillations for the growth. The inset shows the RHEED patterns of STO prior to the growth (left) and LSMO film after the growth on STO (right). (**b**) The typical AFM image. (**c**) An AFM line profile across the terraces that is marked with a line in the Fig. 1(b). (**d**) XRD *θ*-2*θ* scan taken around the (002) peaks of STO and LSMO.

In order to explore the QIEs and localization theory in ultrathin films, we choose LSMO films with a thickness ranging from 20 to 30 unit cell (u.c.). Figure 1(a) shows the *in-situ* reflection high-energy electron diffraction (RHEED) intensity oscillations of the grown film, indicating that the growth proceeds in an ideal 2D layer-by-layer mode^15^. The peaks of the RHEED oscillations represent the growth of exact u.c.-control thickness. The left inset of Fig. 1(a) is the typical RHEED pattern of STO (001) substrate prior to deposition at 750 °C, while the sharp streaky line in the right inset is characteristic of LSMO RHEED pattern after deposition. An atomically smooth surface with clear steps is evidenced in a typical atomic force microscope (AFM) image (1 × 1 μm^2^) [Fig. 1(b)]. An AFM line profile [Fig. 1(c)] across the terraces shows that the average terrace height is about 0.4 nm. The step height corresponds to the lattice constant of LSMO, indicating a well-defined, atomically flat surface. Figure 1(d) presents the x-ray diffraction (XRD) pattern of the LSMO film. Though the film is ultrathin, the peak of LSMO can still be clearly seen, which further proves the single-crystal structure of our high-quality LSMO films.

### Low-temperature resistivity minimum

**Figure 2:**
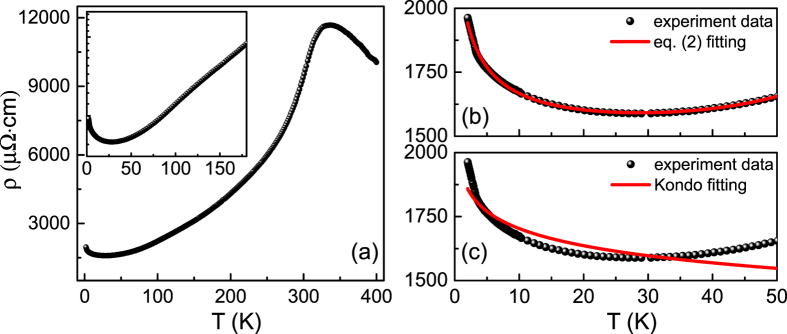
Low-temperature resistivity minima. (**a**) Temperature-dependent resistivity of epitaxial LSMO ultrathin film. The inset is the enlarged low-temperature region in order to visualize the resistivity minimum. (**b,c**) Temperature dependence of resistivity at low temperatures. The solid lines are the fitting results using eq. (2) and Kondo effect, respectively.

Figure 2(a) shows the resistivity versus temperature curves in the range of 2–400 K for the sample (25 u.c.). As the temperature decreases from 400 K in the paramagnetic phase, the resistivity gradually increases and reaches a maximum, indicative of a metal-insulator transition (*T*_p_) at 340 K. The *T*_p_ value is consistent with its Curie temperature (*T*_C_) as determined by extrapolating the measured temperature-dependent magnetization curve. Here *T*_p_ is close to the *T*_C_ of the bulk LSMO (*T*_C_ ~ 369 K^16^), further showing the high quality of our LSMO films. In ferromagnetic phase (*T* < *T*_C_), the LSMO ultrathin film exhibits a typical metallic behavior down to low temperatures. Interestingly, the resistivity does not show a residual resistance behavior for *T*→0, but reaches a minimum at temperature (*T*_min_) around 25 K and increases until the temperature decreases to 2 K, as shown in the inset of Fig. 2(a). The total resistivity in first order correction terms is given by^11^:


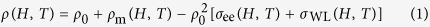


where *ρ*_0_ is the residual resistivity, *ρ*_m_ is the magnetic resistivity contribution from anisotropic MR and magnon scattering, and the *σ*_ee_ and *σ*_WL_ are the conductivity from the contribution of e-e interaction and WL effect, respectively. According to the localization theory and the insensitivity of e-e interaction in strong-correlated manganites, the eq. (1) can be simplified as follows^7^:


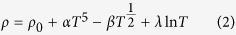


In this equation, *ρ*_0_ is the residual resistivity, *αT*^5^ is the contribution from the inelastic scattering, which is independent of the external fields, *βT*^1/2^ is due to the e-e interaction, and the last term comes from the WL effect. We fit the temperature-dependent resistivity under low temperatures using eq. (2) very well, as shown in Fig. 2(b), indicating that it is the QIEs that give rise to the resistivity minima.

However, Kondo effect should be taken into account as an optional mechanism yielding the resistance upturn. It arises from the exchange interaction between itinerant conduction electrons and localized spin impurities, leading to anomalous temperature dependences in various physical parameters^4^. Kondo effect should contribute to a *ρ* ∝ lg(*T*/*T*_0_) behavior, where *T*_0_ is the Kondo temperature^12^. We then fit the same resistivity versus temperature curve according to 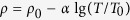, as shown in Fig. 2(c). Apparently, this fitting is not compatible with our experimental data, thus ruling out the Kondo effect as an origin. In addition, another mechanism of spin-polarized tunneling through grain boundaries can be also excluded by the above structural verification of the single-crystal nature (Fig. 1). Moreover, a strong peak of temperature-dependent resistivity is observed near *T*_C_ in single crystals [Fig. 2(a)]. Otherwise, the resistivity of a polycrystalline samples should exhibit a wide maximum at temperature well below *T*_C_, which further rules out the mechanism of tunneling via grain boundaries.

### Electron-electron interaction

**Figure 3:**
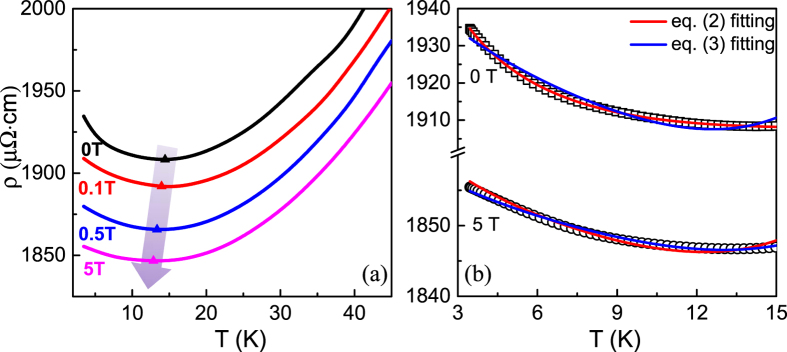
Temperature dependent resistivity under different applied magnetic fields at low temperatures. (**a**) The resistivity upturn is suppressed by the applied magnetic fields, and *T*_min_ shifts towards the lower temperature (see arrow). (**b**) Temperature-dependent resistivity without magnetic field and with high magnetic field (5T), which are fitted using eqs. (2) and (3), respectively. The solid lines are the fitting results. Note that the magnetic field is applied perpendicular to the plane of the film.

The single-crystal LSMO ultrathin film (30 u.c.) is measured at temperatures ranging from 2 to 50 K by applying different magnetic fields to confirm the contribution of e-e interaction to the resistivity minima. Figure 3(a) shows the temperature-dependent resistivity at different magnetic fields. It is seen that the resistivity minima (*T*_min_) still exist under different magnetic fields. *T*_min_ shifts to the lower temperature with increasing magnetic field, in agreement with the previous results^1^,^7^. Such a field-dependent behavior can further rule out the contribution from the Kondo effect and spin-polarized tunneling. Also, the e-e interaction in QIEs is independent of the magnetic field, whereas the WL effect is magnetic-sensitive and can be suppressed under a magnetic field since the field destroys the wave coherence and the self-interference effects are reduced. The low-temperature resistivity upturn under high magnetic fields still exists and can be fitted by *T*^1/2^, where the minimum comes from the e-e interaction alone. Therefore, the experimental data obtained from the high magnetic field (5 T) can be fitted using the following equation without the contribution of the WL effect:


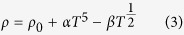


Figure 3(b) shows the comparison of fitting results using eqs. (2) and (3), respectively. It is found that the data without field are better fitted by eq. (2), while the data under 5 T are better fitted by eq. (3). This clearly demonstrates that the resistivity upturn is the result of the combination of the e-e interaction and the WL effect. Table 1 summarizes the fitting results under different magnetic fields. It is seen that even under field of 0.1 T the WL effect is totally suppressed and can be ignored. The presence of e-e interaction in QIEs has been fingerprinted by the *T*^1/2^ dependent resistivity under magnetic fields. At the same time, the existence of e-e interaction under different fields reflects a general characteristic of the strong correlated interaction in the mixed-valent manganites.

### Weak localization effect

**Figure 4:**
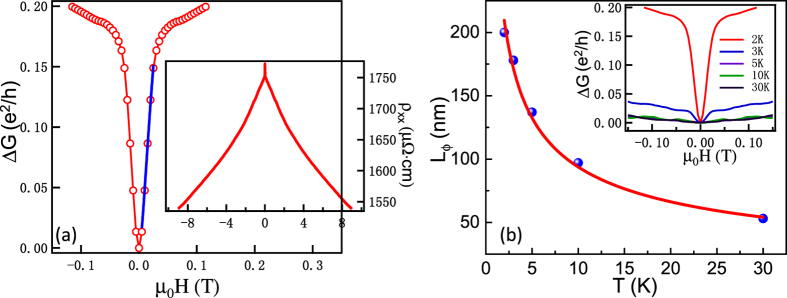
The WL effect in MR measurements of single-crystal LSMO ultrathin films. (**a**) The Δ*G* versus magnetic field curve at 2 K. The solid curve is fitted to the conductance change using eq. (5). The inset is the raw MR. (**b**) Temperature dependence of phase-coherence length. The solid line is the power law fit, which gives *L*_*ϕ*_ ∝ *T*^−1/2^. The inset is the Δ*G* versus magnetic field curves at different temperatures.

The WL effect is revealed by an increase in resistivity at low temperatures and by a peculiar MR behavior related to the phase shift induced by the magnetic field. Although many previous reports^2^,^11^,^12^,^13^,^14^ attributed the resistivity minima to the combination of e-e interaction and WL effect, the direct evidence of the WL effect in MR measurements remains lacking. Figure 4(a) shows the MR or magnetoconductance results at 2 K, where the presence of sharp cusps near zero magnetic field is clearly seen. The similar phenomena have already been evidenced in magnetic topological insulators as well as In_2_O_3_/ZnO thin films^17^,^18^,^19^. According to Maritato *et al*.^2^, a crossover from three-dimensional to 2D behavior of the WL effect takes place when the thickness is below 20 nm. The WL effect in our LSMO ultrathin films is expected to be 2D. Hence, the localization model is given as follows^20^:


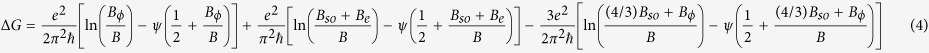


where *ψ* is the digamma function, *B*_*ϕ*_, *B*_*SO*_ and *B*_*e*_ are the phase coherence characteristic field, the spin-orbit characteristic field, and elastic characteristic field, respectively. The corresponding characteristic lengths are deduced from 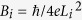 (with *i* = *ϕ*, SO, and *e*), where *L*_*ϕ*_ is the distance traveled by an electron before it loses its phase coherence, *L*_*SO*_ is the length of electron undergoes the effect of the spin-orbit interaction, and *L*_*e*_ is the mean free path. In the limit of strong spin-orbit coupling in correlated materials, the eq. (4) can be reduced to^5^,^21^:


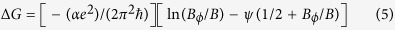


where 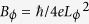. *L*_*ϕ*_ is the phase-coherence length, which describes the quantum correction to the conductivity in the 2D systems. We fit the sheet magnetoconductance change (Δ*G*) by eq. (5) near zero magnetic field where 2D WL effect dominates, yielding *L*_*ϕ*_ ≈ 200 nm at 2 K. Temperature-dependent Δ*G* of the LSMO ultrathin film is shown in the inset of Fig. 4(b), indicating the suppressed WL effect with increasing temperatures.

## Discussion

In order to explore the localization theory in LSMO ultrathin films, and further verify the 2D character of weak localization, the temperature-dependent *L*_*ϕ*_ is depicted. The solid circles in Fig. 4 (b) show the extracted value of *L*_*ϕ*_ and the line is the power law fit of *L*_*ϕ*_ versus temperature. The power law fit gives *L*_*ϕ*_ ∝ *T*^−1/2^, corresponding to the exact 2D localization theory^22^.

In the localization model, *B*_*ϕ*_ is the phase coherence characteristic field. When the magnetic field is larger than *B*_*ϕ*_, the WL effect is suppressed since the field destroys the wave coherence and hence the self-interference effects are reduced. We deduce the *B*_*ϕ*_ = 0.0508 T at 30 K by 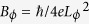. It is suggested that the critical magnetic field should be around 0.05 T under low temperature range, above which the contribution of the WL effect can be ignored. This assumption is consistent with the above fitting results (Table 1), in which when the magnetic field is above 0.1 T, the WL effect is suppressed and the e-e interaction is prevalent in QIEs.

Single-crystal LSMO ultrathin films with atomically flat surface have been epitaxially grown by LMBE. The temperature dependence of resistivity shows generally minima at low temperatures and the *T*_min_ shifts towards the lower temperature with increasing magnetic fields. We give the direct experimental evidences of the e-e interaction and the WL effect responsible for the resistivity minima, thus reaching a solid conclusion that the low-temperature resistivity minima in single-crystal manganites are the result of QIEs due to the combination of the WL effect and the e-e interaction. Moreover, the power law fit of *L*_*ϕ*_ versus temperature further confirms that the WL effect is 2D, which can help to understand the localization theory in strong correlated manganites. Our study may deepen the fundamental understanding of the physical mechanism of the resistivity minimum behavior observed in single-crystal manganites. The high-quality LSMO ultrathin films can serve as a platform for the fabrication of electronic devices for applications in spintronics and energy.

## Methods

### Epitaxial Growth

The LSMO ultrathin films were grown by LMBE technique (KrF excimer laser with *λ* = 248 nm and *E* = 100 mJ). The repetition rate of the laser was 2 Hz with a pulse duration of 20 ns. During the growth *in-situ* RHEED monitor was employed to achieve high-quality epitaxial growth and the thickness was well controlled. Prior to growth, the STO (001) substrates (5 × 5 mm^2^) were chemically etched with HF and then annealed in high-purity oxygen atmosphere for about four hours at 950 °C^23^,^24^. TiO_2_-terminated STO substrates with atomic terraces were hence achieved, as verified by AFM. STO substrates were then ultrasound cleaned in acetone and *in-situ* annealed in 10^−6^ mbar ozone atmosphere at 800 °C for 1 h. With this modified substrate treatment^25^, the RHEED intensity oscillations were readily obtained. The temperature during film growth was maintained at 750 °C. The chamber was firstly evacuated down to 7 × 10^−8^ mbar and then a mixture of oxygen and 20% ozone atmosphere at a pressure of 2 × 10^−3^ mbar was used for the growth to obtain a strongly oxidizing atmosphere. At the end of deposition, the film was cooled down at the growth pressure without further annealing. With the help of ozone^26^, the epitaxial thin films have good quality with no need for post-annealing^27^.

### Structural Characterization

The surface morphology was examined by a high-resolution AFM system (Asylum Cypher under ambient conditions). The crystal structure was examined by a *θ*-2*θ* XRD (Bruker D8 Discover).

### Transport Measurements

The transport properties were measured in a standard four-probe technique by a Quantum Design physical property measurement system (PPMS-9T).

## Additional Information

**How to cite this article**: Niu, W. *et al*. Evidence of weak localization in quantum interference effects observed in epitaxial La_0.7_Sr_0.3_MnO_3_ ultrathin films. *Sci. Rep.* **6**, 26081; doi: 10.1038/srep26081 (2016).

## Figures and Tables

**Table 1:** The *R* ^2^ fitting results under different magnetic fields by eqs. (2) and (3), respectively.

Applied magnetic field (T)	*R*^2^	
	eq. (2)	eq. (3)
0	0.99986	0.97476
0.1	0.9869	0.99162
0.5	0.98822	0.99811
5	0.97738	0.99127
